# Dysphagia Induced by Nasal Septal Chondroma: A Case Report

**DOI:** 10.31729/jnma.3953

**Published:** 2019-06-30

**Authors:** Kang-Duk Suh, Hyun-kyu Chae, Mun-Young Chang, Seog-Kyun Mun

**Affiliations:** 1 Department of Otorhinolaryngology-Head and Neck Surgery, College of Medicine, Chung-Ang University, Seoul, Korea

**Keywords:** *chondroma*, *dysphagia*, *septum*

## Abstract

Chondromas rarely occur in the nasal area and are usually found in the metaphyseal area of the phalanges and metacarpals of the hands, as well as the pelvis, sternum and scapula. The authors present an unusual case of dysphagia induced by histologically confirmed chondroma arising from the nasal septum. Treatment is to completely remove the mass with adequate margins of normal tissues to prevent recurrence and malignancy. Intranasal endoscopic removal of tumor with an adequate margin of normal tissue. After one year of treatment, there was no evidence of recurrence. We present a case of nasal septal chondroma in 18-year-old male.

## INTRODUCTION

In patients with dysphagia, esophagitis or esophageal motility disorders may be common causes, but in rare cases, esophageal stricture, pharyngoesophageal diverticulum, neuromuscular disorder, cricopharyngeal spasm, gastroesophageal reflux disease may be the reason.^1,2^ Also, tumors arising from the hypopharynx, larynx, esophagus, and mediastinum may cause dysphagia.^3^ Of those tumors reported, chondroma is a rare disease causing dysphagia. Chondroma is a benign tumor usually arising from the femur, lung or breast; it rarely occurs in the head and neck region.^4^ We present a case of dysphagia induced by chondroma arising from the nasal septum removed by endoscopic operation.

## CASE REPORT

**Figure 1.**
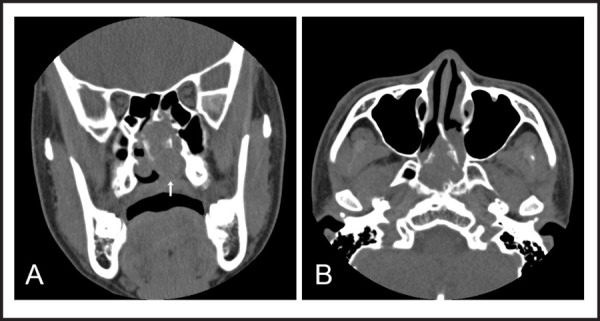
(A) Coronal view of computed tomography scan shows about 4.0 x 3.0 cm heterogeneously enhancing mass which obliterates the nasopharynx and attaches to the basisphenoid and soft palate (white arrow), (B) Axial view of computed tomography scan shows posterior nasal septum which was destructed by the tumor.

**Figure 2.**
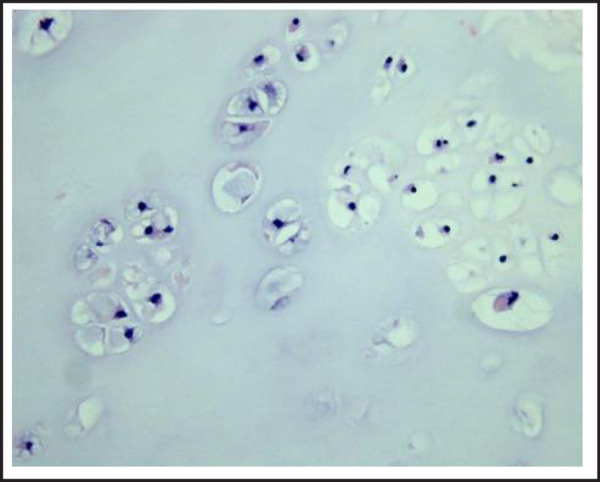
Microscopic finding showing chondrocytes interspersed throughout cartilaginous ground substance (hematoxylin and eosin; x200).

A male patient aged 18-year-old visited our otorhinolaryngology outpatient clinic who was referred to our department from the division of gastroenterology complaining of lump sensation and intermittent dysphagia for the past six months who showed no specific findings in esophagogastroscopy. Dysphagia worsened after eating solid food rather than liquid food, but the patient did not complain of hoarseness, odynophagia and aspiration. The patient had nasal obstruction for two months, but did not complain of any other nasal symptoms. No pharmacologic treatment could lead to improvement of symptoms. The patient suffered facial trauma after falling down the stairs five years ago, but had not received treatment due to minor injuries. Other than that, there were no family history and operation records. On physical examination, the nasal septum was clearly deviated to the right with the right nasal cavity almost completely obstructed. The left nasal cavity was partially obstructed due to concha bullosa and hypertrophy of the uncinate process. Just posterior to these structures, there was a hard circular mass surrounded by normal mucosa completely obstructing the posterior nasal cavity extending to the nasopharynx in adhesion to the floor of the left nasal cavity. Upon swallowing, there was significant loss of mobility of the floor of the left nasal cavity compared to the right due to the adhesion with the mass. Computed tomography (CT) of the sinus showed a non-enhanced, round, homogeneous mass measuring 4.0 x 3.0 cm in the nasal cavity and the nasopharynx with partial bone destruction in the posterior nasal septum and the sphenoid sinus. Also, adhesion between a mass and soft palate was observed (Figure 1A and 1B). Patient agreed to remove nasal cavity mass through surgery after the CT can result, an excision with an endoscopic operation under general anesthesia was performed. Incision was made in front of the mass to approach the septum. After being reported of a possible benign tumor suggestive of chondroma in frozen section, the tumor was completely removed. Gross findings showed a soft, covered mass originating from the posterior nasal septum extending to the nasopharynx and partially destructing the perpendicular plate, posterior vomer and front wall of the sphenoid sinus. The mass was easily removed from its surrounding tissues, but a curette was used to remove the area that was strongly attached to the front wall of the sphenoid sinus. Tumor was removed in piece meal, since the size of the mass was large and there was a hardly attached part, which did not allow en-bloc resection. After removal of the mass, dissector was used to carefully dissect the adhesion in floor of left nasal cavity and packing was done for both nasal cavities. Since nasal septal deviation was not severe, septoplasty was not performed. Histologic findings confirmed it as a well differentiated chondroma composed of hyaline cartilage. Chondrocytes with small, consistent nuclei were found and there were no signs of dysplasia of the nuclei or ossification (Figure 2). The patient's symptoms were relieved and was therefore discharged three days after operation. The patient is currently on follow up with no signs of recurrence for one year. There's no adverse or unanticipated events to patient.

## DISCUSSION

First reported by Morgan in 1842, chondroma was controversial in its pathogenesis and etiology. It is now categorized, however, as a benign bone tumor. It usually arises in the orthopedic area and rarely occurs in the head and neck area.^4^ It may affect all ages but usually occurs in middle-aged people of either gender.^1^ The etiology of chondroma is unclear; there are two suggested theories. One suggests abnormal enlargement due to localized inflammation caused by stimulation of infected chondrocytes.^1^ The other theory involves cartilaginous material interacting with fibrous material in a cyst caused by trauma to the cartilage or the bone.^5^ In this case, trauma seems to play a major role in the pathogenesis of the chondroma, but the etiology remains unclear.

Clinical manifestations of chondromas are incidentally found, as it is usually a painless mass.^1,5^ In cases of chondromas occurring in the nasal cavity, the patient might suffer from nasal obstruction, rhinorrhea, or epistaxis as the size of the chondroma increases. Rarely, swelling of the buccal area and exophthalmosmay develop.^6^ Unfortunately, in this case, diagnosis was delayed since dysphagia was the main symptom rather than nasal symptoms. Diagnosis of chondromas is made with CT or MRI, but in cases arising from the nasal septum, detection may be difficult.^6^ First, since it presents as a painless mass, diagnosis is delayed until the mass is big enough to cause nasal symptoms. Second, chondromas are very rare compared to other diseases that cause dysphagia. Third, it is easy to misdiagnose it as osteoma, ossifying fibroma and fibrous dysplasia from the ground glass opacification found on CT. Lastly, MRI shows inconsistent enhancement with no specific findings. Differentiation should be made from mucous cyst, osteoma, ossifying fibroma, fibrous dysplasia and angiofibroma. Chondromas require long term follow up as these tumors may progress to chondrosarcomas.^7^ Diagnosis is confirmed by histology, and gross findings present with a mass that is smooth and rather firm, with a ripe pear consistency.^4^ Microscopic studies show well differentiated hyaline cartilage and evenly arranged chondrocytes that are similar to normal cartilaginous tissues with no anaplasia.^1^

Treatment is to completely remove the mass with adequate margins of normal tissues to prevent recurrence and malignancy.^8^ If surgical approach is difficult, long term medication such as non-steroidal anti-inflammatory drugs is reported to be beneficial, but surgery is recommended due to risks of recurrence and malignancy such as chondrosarcoma.^7,8^ In the past, extensive excision of the nose or maxilla was done, but currently it is endoscopically removed. The advantages of endoscopic removal with such as the procedure in this case are prompt recovery, made possible due to minimal damage to the mucosa, the ability to correct deviated nasal septum, directly having access to the mass with normal tissues spared, and obtaining satisfactory cosmetic results.

In this case, we suppose that the mass was causing the patient's symptom because it was firmly attached to the soft palate. While the oral phase of swallowing, the soft palate separates the oral cavity and pharynx. Soft palate then rises, separates oropharynx and nasopharynx during the pharyngeal phase of swallowing.^9^ Any loss of palate mobility will affect these functions of the soft palate, it eventually leads to allow pressure and bolus material to escape inappropriately.^9^ In light of the significant decrease in the movement of the patient's left nasal cavity base, it may be assumed that this would have affected the soft palate movement, resulting in dysphagia.

In the presence of dysphagia symptoms, it is necessary to distinguish whether the symptom occurs after swallowing or initiating swallowing to distinguish whether oropharyngeal dysphagia or esophageal dysphagia. Also, to distinguish between neuromuscular disorders such as achalasia and esophageal spasm, as well as mechanical obstructions such as stricture and cancer, doctor should check whether the symptom occurs only in solid food or liquid diet.^10^ However, in this case, it was difficult to diagnose because dysphagia was caused by chondroma in the nose. We would like to present that dysphagia can be a symptom originating from the nose as well as the mouth or esophagus.

## Consent

**JNMA Case Report Consent Form** was signed by the patient and the original is attached with the patient's chart.

## Conflict of Interest

**None.**
